# The Formation Mechanism of (001) Facet Dominated *α*‐FAPbI_3_ Film by Pseudohalide Ions for High‐Performance Perovskite Solar Cells

**DOI:** 10.1002/advs.202300056

**Published:** 2023-04-23

**Authors:** Shengwen Li, Junmin Xia, Zhaorui Wen, Hao Gu, Jia Guo, Chao Liang, Hui Pan, Xingzhu Wang, Shi Chen

**Affiliations:** ^1^ Institute of Applied Physics and Materials Engineering University of Macau Macao Macao SAR 999078 China; ^2^ Department of Materials Science and Engineering Southern University of Science and Technology Shenzhen Guangdong Province 418055 China

**Keywords:** (001) facet, binding energy, DFT calculation, perovskite solar cell, pseudohalide anions

## Abstract

Formamidinium lead triiodide (*α*‐FAPbI_3_) has been widely used in high‐efficiency perovskite solar cells due to its small band gap and excellent charge‐transport properties. Recently, some additives show facet selectivity to generate a (001) facet‐dominant film during crystallization. However, the mechanism to realize such (001) facet selectivity is not fully understood. Here, the authors attempted to use three ammonia salts NH_4_X (X are pseudohalide anions) to achieve better (001) facet selectivity in perovskite crystallization and improved crystallinity. After addition, the (001) facet dominance is generally increased with the best effect from SCN^−^ anions. The theoretical calculation revealed three mechanisms of such improvements. First, pseudohalide anions have larger binding energy than the iodine ion to bind the facets including (110), (210), and (111), slowing down the growth of these facets. The large binding energy also reduces nucleation density and improves crystallinity. Second, pseudohalide ions improve phase purity by increasing the formation energies of the *δ*‐phase and other hexagonal polytypes, retarding the *α*‐ to *δ*‐phase transition. Third, the strong binding of these anions can also effectively passivate the iodine vacancies and suppress nonradiative recombination. As a result, the devices show a power conversion efficiency of 24.11% with a *V*_oc_ of 1.181 V.

## Graphical abstract

Pseudohalide ions can modulate the facets during crystallization, forming a (001) dominated surface. The regulation is due to larger binding energy at the high index facets, inducing preferential growth along (001) direction. Less intrinsic defects are found resulting in PCE of 24.11% and *V*_oc_ of 1.181 V with slightly enhanced stability.

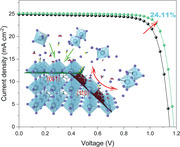

## 1 Introduction

Perovskite solar cells (PSCs) attracted extensive research interest in the past decade due to their outstanding optical and electric properties. Recently, its power conversion efficiency (PCE) has been boosted to 25.7%.^[^ ^1^ ^]^ Metal halide perovskites with the general formula of APbX_3_, where A is a cation mainly using methylammonium (MA, CH_3_NH_3_^+^), Formamidinium (FA, NH_2_CH = NH_2_^+^), and cesium (Cs^+^), and X is halide anion such as Cl^−^, I^−^, and Br^−^, are the key components of these light‐harvesting materials.^[^ ^2^, ^3^, ^4^ ^]^ Compared with MAPbI_3_, the cubic *α*‐phase FAPbI_3_ has a narrower bandgap (1.45–1.51 eV in thin films), showing a higher Shockley‐Queisser limit for the single‐junction photovoltaics.^[^ ^5^, ^6^ ^]^ In addition, FAPbI_3_ exhibits smaller free volume and effective mass than MAPbI_3_, indicating superior carrier transport properties. Therefore, FAPbI_3_‐based single‐junction PSCs have achieved the highest PCE than the other compositions.^[^ ^7^, ^8^, ^9^ ^]^

However, cubic *α*‐FAPbI_3_ is metastable at room temperature and may undergo a phase transition into the undesired nonperovskite *δ*‐phase. Such transition is attributed to the large anisotropic lattice strain and the disorderliness of cation rotation in the *α*‐FAPbI_3_, resulting in a larger formation energy than the *δ*‐FAPbI_3_.^[^ ^10^ ^]^ As a result, the *α*‐FAPbI_3_ film usually contains *δ*‐phase, which both reduce light absorption and retard charge transfer. Hence, many efforts have focused on stabilizing the *α*‐phase to achieve high‐quality and phase‐pure film.^[^ ^11^, ^12^ ^]^ Some researchers attempted to make compositional engineering by mixing with small radius cations, such as Cs^+^, Rb^+^, and MA^+^, to reduce lattice strain.^[^ ^13^, ^14^, ^15^ ^]^ Among them, MAPbBr_3_ with both smaller cations and anions is commonly used in the FAPbI_3_ preparation to maximize *α*‐phase stability. Nevertheless, the addition of MAPbBr_3_ creates new problems, such as phase separation, reduced photo absorption (blue‐shifted absorption edge), and lowered thermal stability. To minimize these problems, researchers have to reduce the addition of MAPbBr_3_ content to 5% or less. Alternatively, Soak et al. stabilized the FAPbI_3_ phase by 3.8% MDACl_2_ and 30% MACl and achieved a very high short‐circuit current density of 26.7 mA cm^−2^ and a certified PCE of 23.73%.^[^ ^16^ ^]^ However, many other molecule additives have shown similar improvements in stability and phase purity, but the detailed mechanism behind them is not fully discussed.^[^ ^17^, ^18^ ^]^

It has been noticed that crystal facets in FAPbI_3_ also have a strong influence on film instability and device efficiency. Calculations suggest that the (110) facet possesses a high density of dangling bonds, and the (111) facet is the least stable.^[^ ^19^, ^20^ ^]^ In contrast, the (001) facet with nearly no dangling bonds is the most desired facet for perovskite devices. There are already some reports that used long‐chain alkylamine cations to promote the growth of the (001) facet during crystallization, such as PEAI^[^ ^21^ ^]^ and OAm,^[^ ^22^ ^]^ and show improved performance. But the long‐chain alkylamine cations may block charge transfer and cause inferior PCE in the device.^[^ ^23^, ^24^ ^]^ Generally, the mechanism behind this was proposed due to the changing facets' growth rate after the absorption of ions. Recently, pseudohalide anions such as thiocyanate (SCN^−^),^[^ ^6^ ^]^ formate (HCOO^−^),^[^ ^7^, ^25^ ^]^ and acetate (CH_3_COO^−^)^[^ ^26^ ^]^ have also been reported to slow down the crystallization dynamics and improve (001) facet dominance with less residue. Therefore, they could be a better choice for (001) facet selectivity. One MD simulation study showed that the presence of a low formation energy intermediate phase between *δ* phase and *α* phase by SCN^−^ might explain their high *α* phase purity and stronger (001) facet dominance.^[^ ^6^ ^]^ However, the detailed mechanism of the facet selectivity by additives is not clear, and the effectiveness of this selectivity is far from optimized. A fundamental understanding of the phase transition kinetics and facet formation energies of FAPbI_3_ perovskite has yet to be studied.

In this paper, we combined small cations NH_4_^+^ and three types of pseudohalide anions X^−^ as the additives (X^−^ includes thiocyanate (SCN^−^), formate (Fo, HCOO^−^), and acetate (Ac, CH3COO^−^)) to enhance the facets selectivity in *α*‐FAPbI_3_ film. The NH_4_^+^ is chosen due to its positive effect in crystallization with minimum residue, allowing us to see the real contribution of anions. All three additives provoke (001) facet formation with larger crystallites, resulting in a high‐crystalline and (001) facet‐dominated film. Among them, the SCN^−^ anions show the strongest (001) facet dominance. By first‐principle calculation, we reveal three mechanisms to explain such improvements. First, these pseudohalide salts nearly double the formation energies of the hexagonal polytypes (2H (*δ* phase), 4H, and 6H) of FAPbI_3_, reducing the presence of these phases in the film. Second, pseudohalide anions are found to have larger binding energies on the surface of (110), (111), and (210) facets than on the (001) facet. Therefore, the growth of these facets is significantly suppressed by the strong binding of ions. Our XRD measurements confirm the improved dominance of the (001) facet by the much stronger (001) peak after using the additives. Third, these anions can also strongly bind to the iodine vacancies, effectively reducing the predominant iodide vacancies in FAPbI_3_ films. The reduced iodide vacancies are also beneficial for better crystallinity during growth. Among three pseudohalide anions, the sizes of thiocyanate and formate are smaller than the acetate and can fit into the iodide vacancy sites, giving better passivation effects. As a result, high‐quality FAPbI_3_ perovskite films are grown with improved crystallinity and predominately (001) facets, giving a champion device with 24.11% of PCE with a *V*_oc_ as high as 1.181 V.

## 2 Results and Discussion

**Figure 1:**
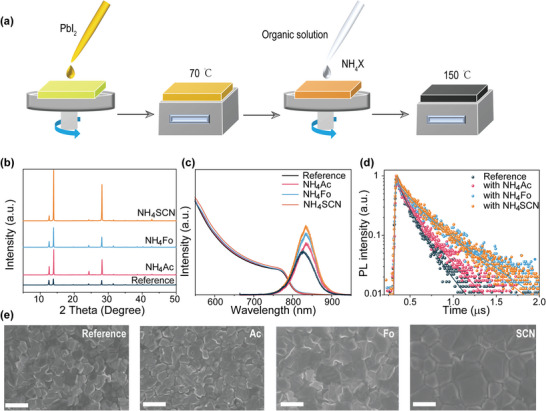
Characterization of FAPbI_3_ perovskite film. a) Simplified scheme presenting the additive engineering of sequential two‐step for FAPbI_3_ perovskite films. b) XRD spectra, c) UV–vis absorption and PL spectra, d) Time‐resolved photoluminescence, and e) Top‐view SEM images of the FAPbI_3_ perovskite films with or without additives.

**Figure** **1**a illustrates the procedures of the sequential two‐step method used in our perovskite film preparation. The pseudohalide anions were added to the organic solution and spin‐coated onto the obtained PbI_2_ films. As a standard protocol, MACl is also added in all samples to stabilize the FAPbI_3_ phase. The as‐fabricated dark brown film was annealed to transfer into the black *α*‐FAPbI_3_ phase. Figure 1b shows the X‐ray diffraction (XRD) spectra of FAPbI_3_ perovskite films with or without NH_4_X. The (001) diffraction peaks of the doped films are all increased while the full widths at half‐maximum of the peaks are significantly reduced (Figure S1, Supporting Information), indicating improved crystallinity with predominated (001) surface orientation. Also, the peak at 12.7° from unreacted PbI_2_ is significantly decreased with NH_4_SCN and NH_4_Fo, except for NH_4_Ac. The larger Ac anion may interfere the ion exchange process, causing less complete PbI_2_ conversion. The reduced PbI_2_ peak suggests a more complete conversion to FAPbI_3_, which can be explained by the formation of metaphase (NH_4_PbI_3_) by NH_4_^+^ cation. The metaphase slows down the crystallization rate, which allows longer intercalation time for FA, resulting in such improvement.^[^ ^27^, ^28^ ^]^ As known, the unreacted PbI_2_ may affect the stability of PSCs and cause larger hysteresis.^[^ ^29^, ^30^, ^31^ ^]^ Therefore, such reduction is beneficial to improve device performance. Figure 1c shows the ultraviolet–visible (UV–vis) absorption spectra and the photoluminescence (PL) spectra of the reference and doped FAPbI_3_ films. The adsorption onset and PL peak position in wavelength are longer in the doped films. The band gap of the doped films decreases from 1.544 to 1.538 eV (Figure S2, Supporting Information). The small band gap reduction is due to reduced MA content in the obtained films. Moreover, the doped films have stronger absorption and increased PL intensities compared with the reference sample, suggesting lesser recombination. Figure 1d shows the time‐resolved photoluminescence (TRPL) of the FAPbI_3_ perovskite films. The doped FAPbI_3_ films show a slower decay than the reference sample, demonstrating decreased nonradiative recombination rate after pseudohalide anions passivation. The perovskite films with smaller pseudohalide anions (SCN^−^ or Fo^−^) exhibit longer carrier lifetimes (*τ*) (SCN^−^, *τ* = 578 ns; Fo^−^, *τ* = 549 ns), while the large anion (Ac^−^, *τ* = 487 ns) has a similar lifetime with the reference film (*τ* = 462 ns).^[^ ^32^ ^]^ From electrochemical impedance spectroscopy (EIS) measurement (Figure S3, Supporting Information), the recombination resistance of SCN‐doped film is the highest, suggesting the least interfacial recombination. The difference in carrier lifetimes could be partially explained by the steric effect of ions where SCN^‐^ and Fo^−^ are small enough to fit into iodine vacancies but Ac^−^ is too large to occupy the sites effectively (Figure S4, Supporting Information). The scanning electron microscope (SEM) top‐view images are shown in Figure 1e. Compared with the reference film, both Fo‐doped and SCN‐doped films show increased average grain sizes. The SCN‐doped samples have the largest average domain size and the most uniform size distribution of domains, which is consistent with its best crystallinity from XRD observation.^[^ ^33^ ^]^ The increased domain sizes suggest that pseudohalide anions change the crystallization dynamics. They could strongly bind to the nucleation sites, and reduce the active nucleation sites in crystallization, resulting in larger average domain sizes. The increased average domain size was also observed in our previous study when nucleation site density is reduced by lower interface passivation.

**Figure 2:**
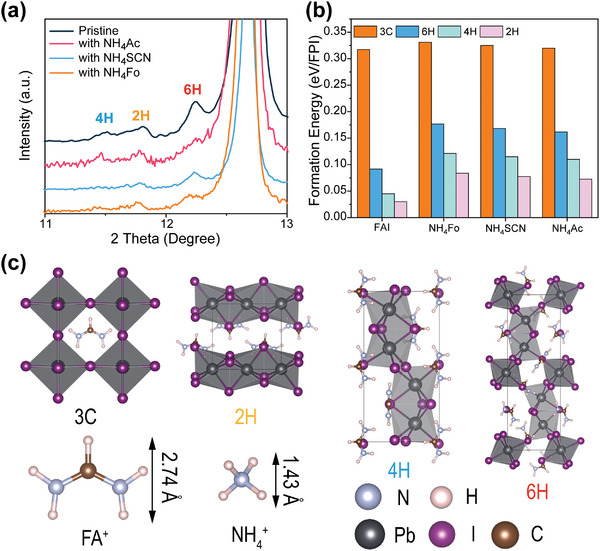
a) Zoom‐in XRD spectra of perovskite thin films with different additives. The three types of hexagonal polytypes are shown next to the PbI_2_ peak. b) The formation energy per FAPbI_3_ primitive cell (FAPI) for the different FAPbI_3_ polytypes, including 3C (cubic phase), 2H (*δ* phase), 4H, and 6H. And c) Corresponding representations of the refined single‐crystal structure viewed along a‐axis.

In the reference sample, a small number of hexagonal polytype phases are observed in the XRD spectrum at 11.6° (4H), 11.8° (2H, *δ*), and 12.2° (6H) (**Figure** **2**a).^[^ ^10^, ^34^ ^]^ The formation of these hexagonal polytype phases is probably related to their low formation energies. However, these hexagonal polytypes are suppressed by these additives. To reveal the mechanism of this suppression, the calculation of the formation energies with and without NH_4_X additives in the lattice is calculated. Without additives, the formation energies per FAPbI_3_ unit cell (FAPI) for 6H, 4H, and 2H polytypes are 0.09, 0.05, and 0.03 eV/FAPI, respectively (Figure 2b). However, the formation energy of cubic phase (3C, *α*) is as high as 0.32 eV/FAPI. The larger formation energy of the 3C phase explains the presence of hexagonal phases at room temperature. When NH_4_X additives are introduced, one FAX salt is replaced by NH_4_X in the minimum volume of the corresponding phase. The formation energies of such lattice are then calculated using the following formula:

(1) $$\Delta E_{f}=\frac{E_{\mathrm{bulk}}-nE_{{\mathrm{PbI}}_{2}}-nE_{\mathrm{FAI}}+E_{\mathrm{FAX}}-E_{{\mathrm{NH}}_{4}X}}{n}$$

From Figure 2b, the formation energies of the 3C phase only slightly increased (<3% increase) with any of the three pseudohalide anions, but the formation energy increases of the hexagonal polytype phases are much more obvious (>80% increase for 6H polytype and >150% increase for 2H and 4H polytypes). The increased formation energies greatly reduce their presence with the additives.

**Figure 3:**
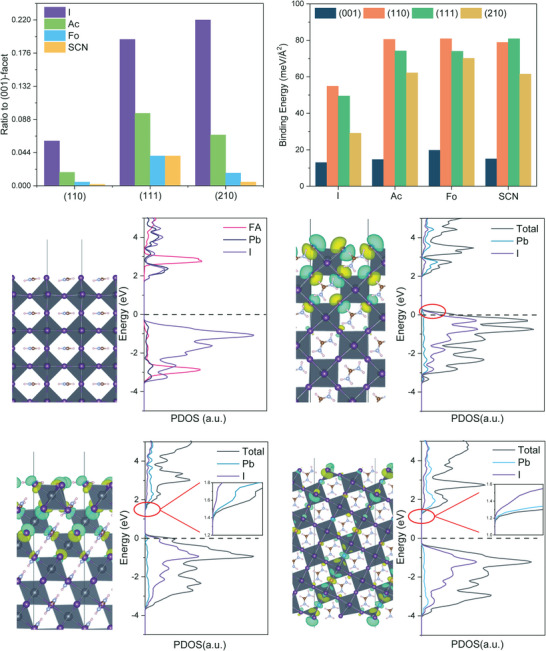
a) The ratios of (110), (111), and (210) diffraction peaks with the (001) peak with different additives. b) The binding energies of various pseudohalide anions on different FAPbI_3_ facets. For the iodine anions, the binding energy is equal to the formation energy of each facet. c–f) Defect states and PDOS of FAPbI_3_ slabs with different surface facets, and the inset picture highlight the surface defects circled by the red line.

The most significant effect of NH_4_X additives is to control the facets of perovskite film during crystallization. From the X‐ray diffraction pattern, the ratio of different facets to (001) facet is summarized in **Figure** **3**a. It is obvious that the ratios of (110), (111), and (210) facets are significantly reduced after the addition of NH_4_X additives. Therefore, an enhanced (001) facet selectivity is shown by three NH_4_X additives. Among them, SCN^−^ shows the best reduction effect. To understand the enhanced (001) facet selectivity, we calculated the binding energy of these pseudohalide ions to different facets. Similarly, an obvious binding energy increase on (110), (111), and (210) surfaces are observed (Figure 3b). Compared with facets terminated by the iodine ions, pseudohalide ions show larger binding energies (ranging from 24 to 43 meV Å^−2^). However, on the (001) surface, the binding energies of Ac^−^ and SCN^−^ remain almost unchanged (only increased by about 2 meV Å^−2^), and a small increase is seen for Fo^−^ ion (about 6 meV Å^−2^). The larger binding energies suggest that anions can compete with iodine ions to terminate the (110), (111), and (210) facets and retard their growth, while the smaller binding energy makes the growth of the (001) facet easier. Therefore, the increased binding energy of additive ions to the (110), (111), and (210) facets is the key to its facet selectivity on the (001) facet.

The enhanced (001) facet selectivity also reduces intrinsic defects associated with different facets. To examine the intrinsic defects states in different facets, the projected density of states (PDOS) of different facets are calculated in Figure 3c–f. From Figure 3c, the valence band maximum (VBM) of FAPbI_3_ facets is mainly composed of I*‐5p* orbital, and the conduction band minimum (CBM) is composed of Pb*‐6s*, Pb*‐6p*, and I‐*5p* orbitals, consistent with previous reports.^[^ ^22^ ^]^ It can be seen that the VBM and CBM of different surfaces are independent of organic ions (FA^+^). However, the surface defects do have a surface dependence. On (110), (111), and (210) facets, intrinsic defect states are observed due to the unsaturation Pb–I bond (iodine vacancies) at the surface and irregular octahedral arrangement. But no such defect states are found on the (001) facet. Thus, the increased (001) facet dominance also reduces defect density in FAPbI_3_ films. Furthermore, the remaining vacancies on the residual high index facets can also be passivated by strongly bound pseudohalide.^[^ ^35^ ^]^

**Figure 4:**
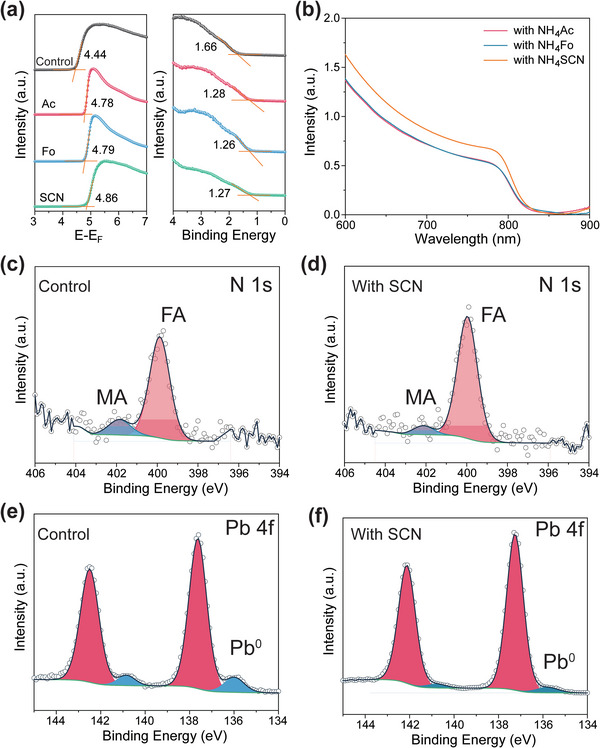
a) UPS of perovskite thin films with different additives. b) UV–vis absorption with different additives. The XPS spectra of: c,d) N 1s and e,f) Pb 4f for films with and without NH_4_SCN additive.

The reduced defect states can be witnessed by the change in doping level in FAPbI_3_ film. The electronic structure of the films with NH_4_X was measured by UV photoelectron spectroscopy (UPS; **Figure** **4**a). The work functions (WF) were determined by the secondary electron cutoff, and they shifted from 4.44 to 4.86 eV with different pseudohalide anion additives (up to 420 mV). Similarly, the valance band maximum (VBM) shifted from 1.66 to 1.26 eV (up to 400 mV). The consistent shifts in VBM and WF suggest reduced n‐type doping after the addition of NH_4_X additives. From the calculation above, we have identified that the n‐type doping is mainly from the I^−^ vacancy in FAPbI_3_. Therefore, the reduced n‐type doping is evidence of reduced I^−^ vacancies. Such reduction could be due to the reduced contents of (110), (111), and (210) facets during crystallization, which have intrinsically less iodine vacancies (Figure 3). It could also be partially attributed to the strong binding of pseudohalide anions to these facets, which effectively passivate the iodine vacancies. Furthermore, the strong bonding of pseudohalide anions also suppresses the formation of Pb_0_. From Pb *2p* spectra in Figure 4e,f, the peak from Pb_0_ is greatly reduced when SCN^−^ is added. The reduced Pb_0_ signal also helps to reduce recombination and supports the longer TRPL lifetime. From Figure 4b, when film thickness was kept the same, the perovskite film with NH_4_SCN shows higher absorption than the films with NH_4_Fo and NH_4_Ac, suggesting better film uniformity and less internal scattering loss. Moreover, the residual of MA^+^ in the SCN^−^doped film is less than the reference^[^ ^36^ ^]^ (Figure 4c,d), which is consistent with the UV results in Figure 1c. Furthermore, from the XPS result (Figure S9, Supporting Information), no recognizable signals from the additives can be found, suggesting that the additive is either completely removed or with any trace amount. However, the trace amount of additive residues may still play a role in passivation, which is an open question requiring further studies.

**Figure 5:**
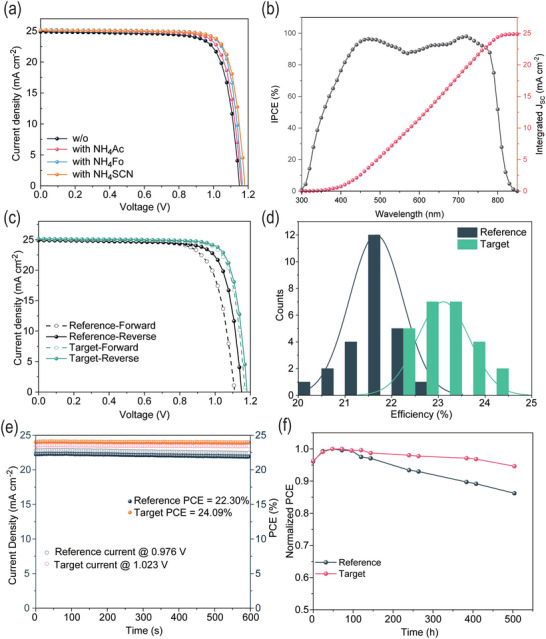
a) *j*‐V characteristics of FAPbI_3_ devices with the addition of three pseudohalide anions salts, and b) IPCE of the devices. c) The hysteresis of device performance with and without NH_4_SCN, d) performance statistics of 25 devices, e) Max power point (MPP) tracking of the devices, and f) Stabilities test in a nitrogen environment.

It can be observed that the crystallinity and (001) facet purity of perovskite film is significantly improved after NH_4_X addition, which should result in better device performance. To prove it, we fabricated PSCs with the structure of ITO/SnO_2_/FAPbI_3_/spiro‐OMeTAD/Au and measured their photovoltaic parameters. **Figure** **5**a shows the typical *j*‐V curves of FAPbI_3_ devices with NH_4_X of three pseudohalide anions. The reference device shows a PCE of 22.43% with a *V*_oc_ of 1.149 V and a fill factor (FF) of 78.43%. The performance of the device with NH_4_Ac was slightly enhanced to 23.27% with a *V*_oc_ of 1.161 V and an FF of 79.75%. The device with NH_4_Fo shows a 19 mV increase of the *V*_oc_ reaching 1.168 V and a better FF of 81.64%, giving a higher PCE of 24.00%. The device with NH_4_SCN shows the largest improvement in PCE, with 32 mV *V*_oc_ enhancement (reaching 1.181 V) and reaches 24.11% in PCE with a FF of 81.23%. This performance enhancement confirms the effectiveness of facet engineering and defects passivation by NH_4_X additives. This is also consistent with the trend of film quality by TRPL shown in Figure 1d and EIS result in Figure S3 (Supporting Information). As shown in Figure 5c, the best device with NH_4_SCN also has lower hysteresis than the reference due to the passivation of iodine vacancies. Better film crystallinity and purer facets also benefit its stability. When the devices with and without NH_4_SCN both showed stable PCE output for 600 s at 24.09% and 22.30%, respectively (Figure 5e), the reference devices lost about 15% of their initial PCE while the doped devices only lost about 5% of their initial PCE after 500 h storage. This stability improvement is also observed with continuous light illumination. Under 1‐Sun condition, the device with NH_4_SCN additive maintained more than 80% of its initial efficiency after 100 h while the reference device dropped to less than 80% (Figure S8, Supporting Information). Our device data prove that good film crystallinity and high (001) facet purity is the key to high‐performance PSCs.

## 3 Conclusions

In summary, we discover that the ammonia salts NH_4_X with pseudohalide anions can significantly improve crystallinity and create a (001) facet‐dominant perovskite film. The mechanism behind this can be attributed to the higher binding energy of pseudohalide anions on the (110), (111), and (210) facets than on the (001) facet. The large binding energy retards the growth of these facets except the (001) facet. As a result, a film with (001) facet dominance is obtained. Meanwhile, the presence of anions also reduces nucleation site density and slows down the crystallization process, resulting in larger and uniform perovskite grains. The smaller ammonia cations can assist the intercalation of FA ions, reducing the presence of unreacted PbI_2_. In addition, the presence of pseudohalide anions also increased the formation energy of hexagonal polytypes, improving the phase purity of the FAPbI_3_ film. Benefiting from the dominance of less defective (001) facet and lesser intrinsic iodine vacancies, the device PCE was clearly improved beyond 24%. Among the three pseudohalide anions, the SCN^−^ and Fo^−^ has smaller size and larger binding energy than the Ac^−^, giving them better device performance. The best PCE is obtained from a device with the addition of SCN^−^, reaching 24.11% and with the highest *V*_oc_ of 1.181 V. Our study reveals a facile method to prepare perovskite films with high crystallinity and dominant (001) facet. We further highlight the importance of the binding energy of additive ions in perovskite crystallization. The large binding energy difference can selectively suppress the defective facets and reduce halide vacancies. By tuning the binding energy by additives, we believe that our strategy has the potential to further improve the performance of PSCs.

## Conflict of Interest

The authors declare no conflict of interest.

## Author Contributions

S.L. and J.X. contributed equally to this work. **Shengwen Li**, Conceptualization, Methodology, Investigation and Writing –Original Draft. **Junmin Xia**, Methodology, and Validation. **Zhaorui Wen**, Characterization on morphology measurement. **Jia Guo, Chao Liang, and Hao Gu**: Characterization on the optical properties and performance measurement. **Xingzhu Wang and Hui Pan**, Validation and resources. **Shi Chen**, Conceptualization, Writing – Review & Editing, Supervision and funding acquisition.

## Supporting information

Supporting Information

## Data Availability

The data that support the findings of this study are available from the corresponding author upon reasonable request.
