# Supplementary material for: The Formation Mechanism of (001) Facet Dominated α‐FAPbI3 Film by Pseudohalide Ions for High‐Performance Perovskite Solar Cells
Source: Adv Sci (Weinh). 2023 Apr 23;10(18):2300056. doi: 10.1002/advs.202300056 (PMC10288232; doi:10.1002/advs.202300056)
Supplement: Supplementary file 1 — Supporting Information [file ADVS-10-2300056-s001.pdf]

Supporting information

The Formation Mechanism of (001) Facet Dominated α-$FAPbI_3$ film by Pseudo-halide Ions for High-Performance Perovskite Solar Cells

Shengwen Li[1+], Junmin Xia[1+], Zhaorui Wen[1+], Hao Gu[1], Jia Guo[1], Chao Liang[1], Hui Pan[1], Xingzhu Wang[2], Shi Chen[1]*

[1]Institute of Applied Physics and Materials Engineering, University of Macau, Macao SAR, China

[2]Department of Materials Science and Engineering, Southern University of Science and Technology, Shenzhen 418055, Guangdong Province, China

E-mail:shichen@um.edu.mo

* Corresponding author

[+]These authors contributed equally to this work

Materials and methods. The precursor materials Lead iodide (PbI_2, 99.999%), Formamidinium iodide (FAI), Spiro-OMeTAD (99.8%), Methylammonium chloride (MACl), bis(trifluoromethane) sulfonimide lithium salt (Li-TFSI, 99%), and 4-tert-butylpyridine (TBP, 96%), Dimethylformamide (DMF, 99.9%), dimethyl sulfoxide (DMSO, 99.5%), Acetonitrile (ACN, 99.9%), isopropanol (IPA, 99.99%), chlorobenzene (99.9%) and Ammonium salts were purchased from Sigma–Aldrich. SnO_2 aqueous solution (tin (IV) oxide, 15% in H_2O colloidal dispersion) was purchased from Alfa Aesar.

Device fabrication. The ITO substrate was cleaned by sequentially sonicating in detergent solution, DI water, acetone, and IPA for 15 min each. A diluted SnO_2 colloidal solution (2.14%, diluted by deionized water) was deposited on the substrate at 3000 rpm for 30 s and annealed at 150 °C for 30 min in an ambient atmosphere. 10 mM KCl was spun-coated at 3000 rpm for the 20s. Then the substrates were annealed at 100 °C for 10 min. Before perovskite coating, the substrates were cleaned by plasma treatment for 15 min. The perovskite solution was prepared by following steps. First, 1.5M PbI_2 was dissolved in a mixed solvent of DMF and DMSO (1 mL, volume ratio at 94:6). After complete dissolution, the PbI_2 solution was filtered through a 0.22 μm pore polytetrafluoroethylene filter and then was spun-coated onto the as-prepared SnO_2 substrates at 1800 rpm for 30 s successively. Then, the PbI_2 film was annealed at 72 ^{o}C for 1 ~ 2 min. Second, the mixed cations isopropanol solution (75 mg FAI, 18 mg MACl, and 1.5 mg NH_4X dissolved in 1 mL isopropanol) was spin-coated onto the PbI_2 layer at 2,000 rpm for 30 s, followed by annealing at 150 ^{o}C for 15 min in the ambient

environment (about 40% relative humidity). Third, the HTL solution was prepared by dissolving 50 mg spiro-OMeTAD in 0.55 ml CB doped by 11.5 μL Li-TFSI (520 mg/mL in Acetonitrile), 5 μL FK209 (375mg/mL in Acetonitrile), and 19.5 μL tBP. The mixed spiro-OMeTAD solution was spun-coated on the surface of the perovskite at 4000 rpm for 30 s. Finally, the top gold electrode was thermally evaporated onto the spiro-OMeTAD layer. The thickness of the gold electrode was adjusted to 100 nm, and the evaporation speed was adjusted to 0.02 nm s^{-1} at the first 20 nm and 0.08 nm s^{-1} for the rest of 80 nm.

Characterization. The solar cell was measured in the glove box with a Keithley 2420 source meter and 425 W collimated Xenon lamp (Newport). The light intensity was calibrated by a silicon cell with 1 sun by 100 mW/cm^2. The XRD spectra of perovskite films were characterized by a Rigaku (RINT-2500) X-ray diffractometer (Cu Ka radiation, λ=1.5418 A), operating at 45 kV and 200 mA over the angular range of $5° \leq 2\theta \leq 60°$. Morphological images were depicted by a field emission scanning electron microscope (JEM-7500F). UV–vis–NIR fluorescence spectrophotometer absorption spectra were performed by a Shimadzu UV 3600 spectrophotometer at room temperature. XPS measurements were carried out with a Thermofisher ESCA lab Xi+, using a monochromatic Al Kα source (1486.7 eV). The XPS spectra were collected at 30 eV pass energy. The UPS radiation was raised by a He-gas discharge lamp (He Iα at 21.22 eV) and were collected at 3 eV pass energy with -5 V bias voltage. Both XPS and UPS were measured by a hemisphere analyzer under a base pressure of 1.0×10^{-8} mbar. PL (excitation at 450 nm) was performed with the Hamamatso spectrometer. The

TCSPC was performed at room temperature. The excitation source was a pulsed ultraviolet picosecond diode laser operating at 405 nm. The signal was dispersed by a 320 mm monochromator (iHR320 from Horiba, Ltd.) combined with suitable filters and detected based on the time-correlated single-photon counting technique.

First-principles calculation. The electronic structures were studied using the Vienna Ab initio Simulation Package (VASP) with the projector augmented wave (PAW) method.[1–3] The generalized gradient approximation functional with the revised Perdew–Burke–Ernzerhof formulation was adopted to describe the exchange-correlation interaction among electrons.[4,5] An energy cutoff of 450 eV was used for the plane wave expansion. $FAPbI_3$ Slabs with about 15-Å thickness and a 15-20 Å vacuum layer along the z-direction were used to model the surface.

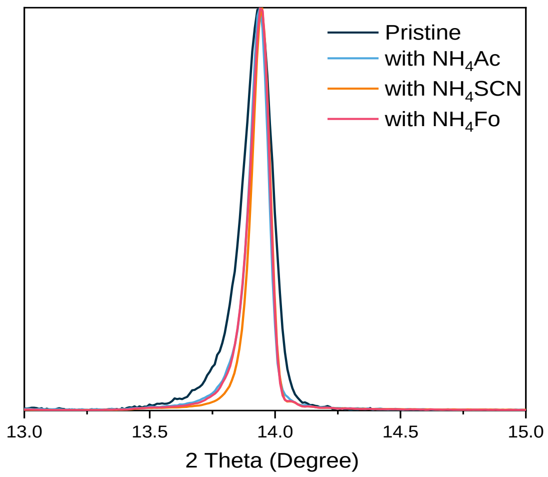

Supplementary Figure 1. XRD data of (001) peaks for perovskite films.

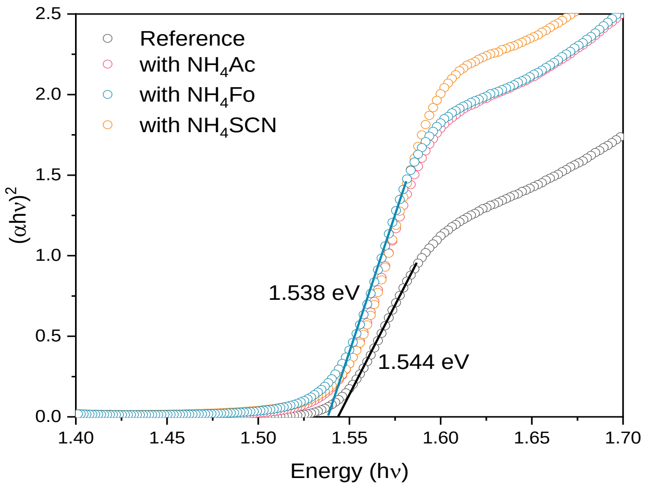

Supplementary Figure 2. Calculation of optical band gap from UV-Vis-NIR absorption spectra.

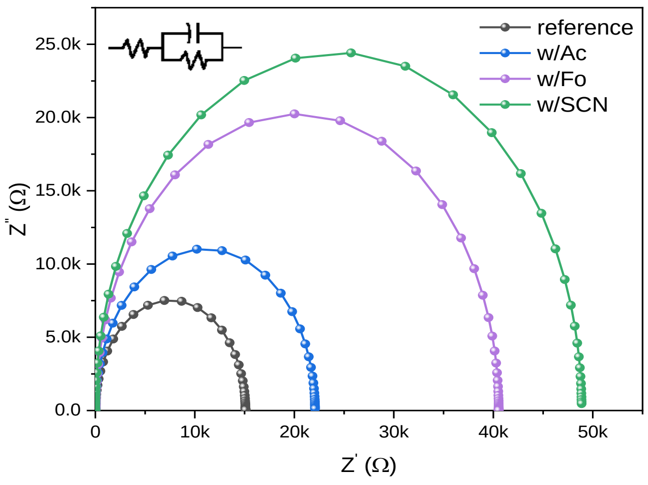

Supplementary Figure 3. Nyquist plots (measured in the frequency range from 1 MHz to 1 Hz at 0 V bias under dark).

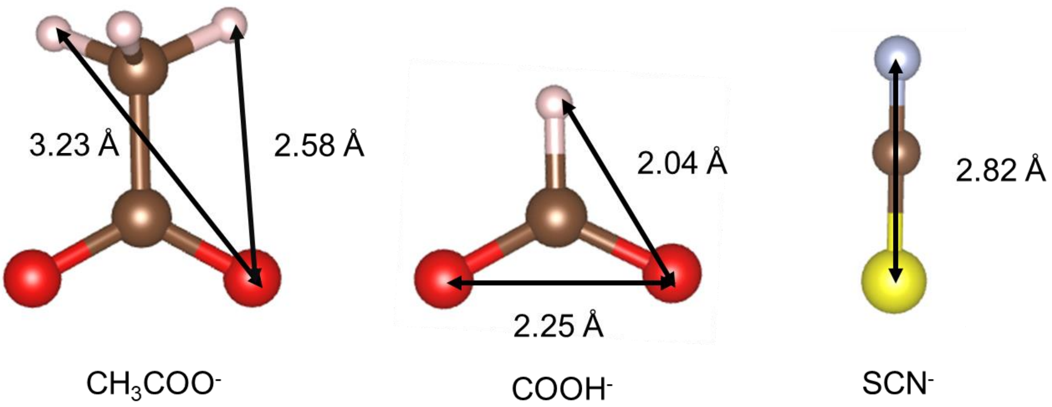

Supplementary Figure 4. Pseudo-halide anion structure and size.

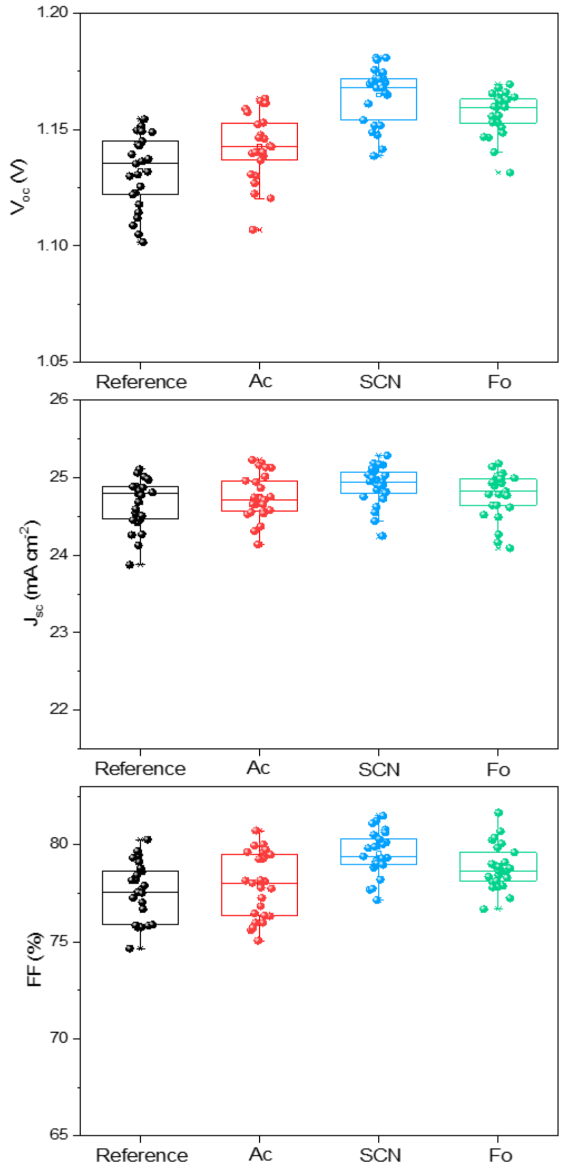

Supplementary Figure 5. Statistical box charts for the photovoltaic parameters of V_{OC}, J_{SC}, and FF of devices without and with NH_4X doping.

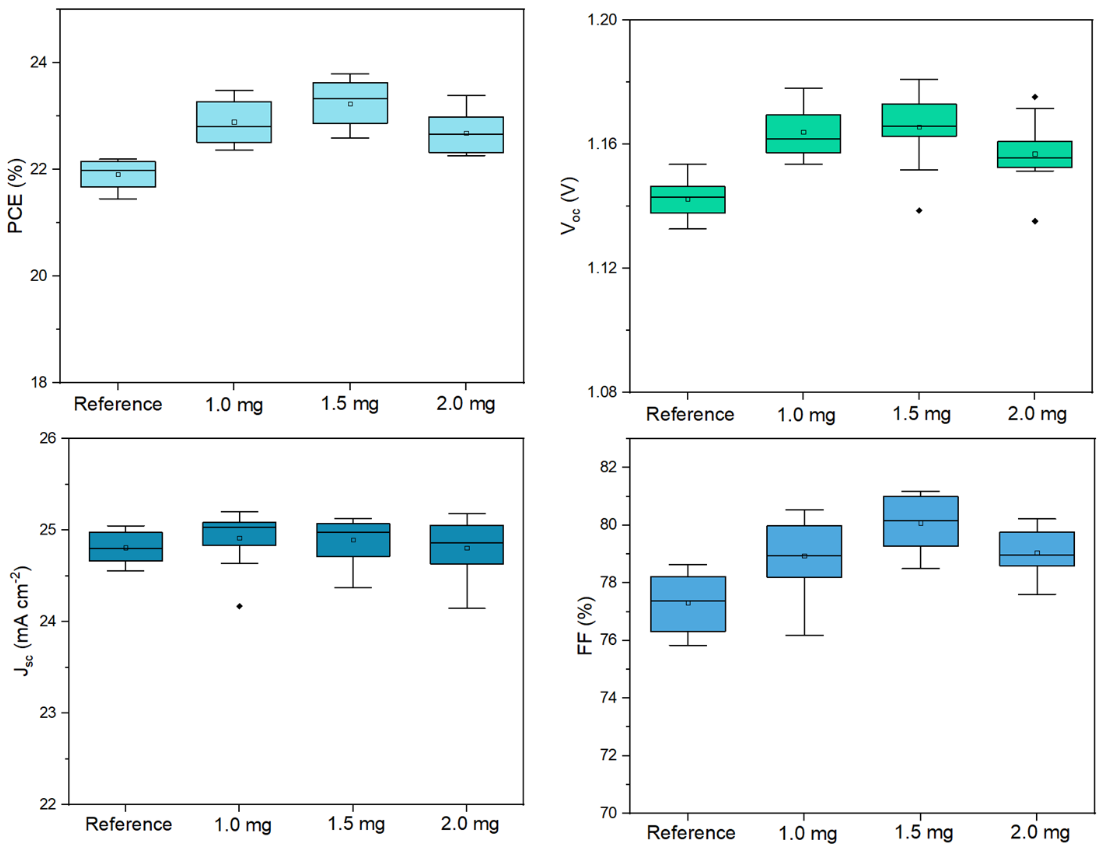

Supplementary Figure 6. Statistical box charts for the photovoltaic parameters of V_{OC}, J_{SC}, and FF of devices without and with different concentration of NH_4SCN doping.

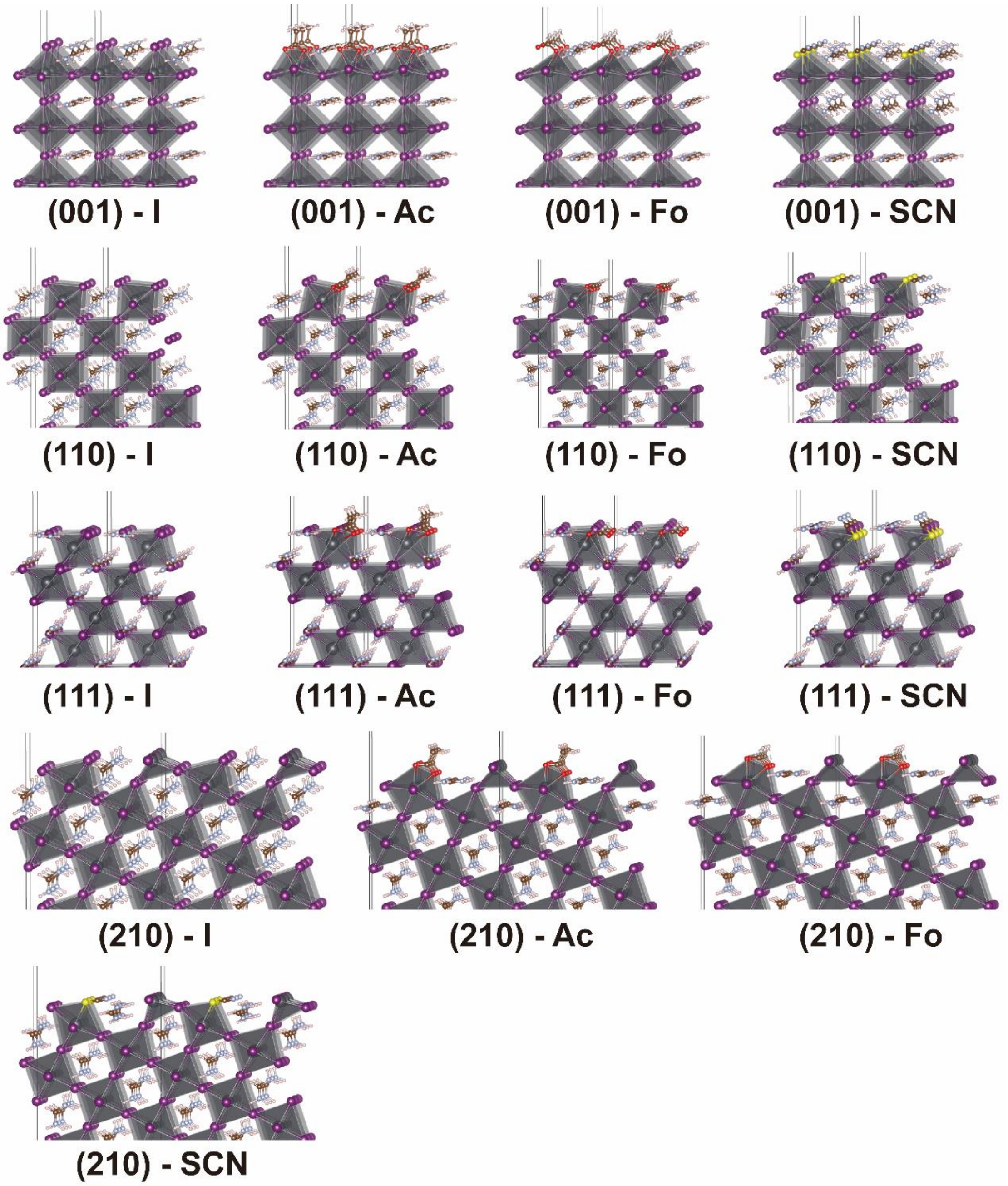

Supplementary Figure 7. Structures of different $FAPbI_3$ facets with different anions.

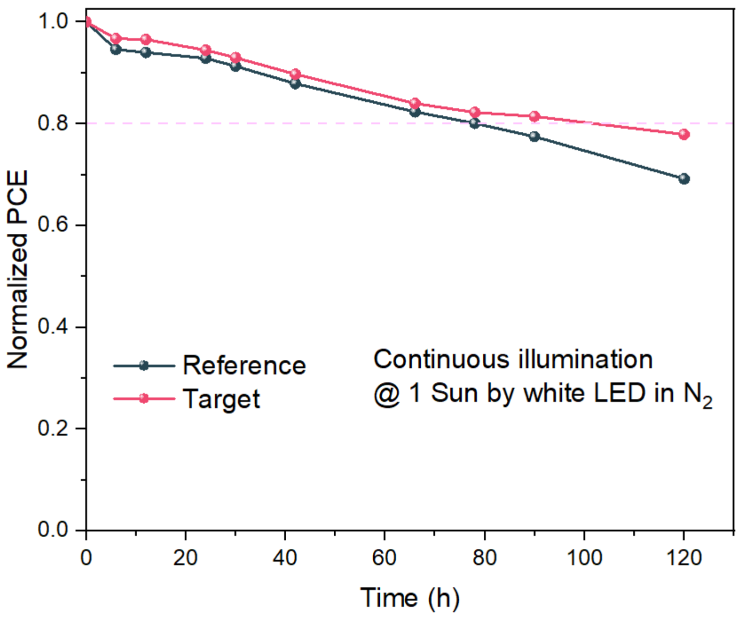

Supplementary Figure 8. Normalized PCE of reference and target devices which are tested under continuous illumination at 1-Sun illumination in an N_2-filled glove box.

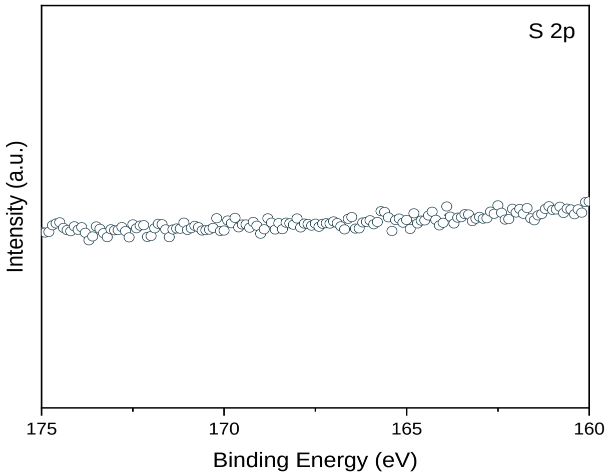

Supplementary Figure 9. S 2p XPS of perovskite with SCN doped.

References:

[1] G. Kresse, J. Furthmüller, *Comput. Mater. Sci.* **1996**, *6*, 15.

[2] G. Kresse, J. Furthmüller, *Phys. Rev. B* **1996**, *54*, 11169.

[3] J. P. Perdew, K. Burke, M. Ernzerhof, *Phys. Rev. Lett.* **1996**, *77*, 3865.

[4] G. Kresse, D. Joubert, *Phys. Rev. B* **1999**, *59*, 1758.

[5] Blöchl P.E., *Phys. Rev. B* **1994**, *50*, 17953.
